# Biomimetic Chromatography Profiling of a Neurotoxicology-Relevant Compound Panel: A Physicochemical Dataset for New Approach Methodologies

**DOI:** 10.3390/biomimetics11090620

**Published:** 2026-09-02

**Authors:** Wiktor Nisterenko, Yash Raj Singh, Katarzyna Ewa Greber, Karolina Jagiełło, Krzesimir Ciura

**Affiliations:** 1 Department of Physical Chemistry, Medical University of Gdansk, Al. Gen. J. Hallera 107, 80-417 Gdansk, Poland; wiktor.nisterenko@gumed.edu.pl (W.N.); yashr.singh@gumed.edu.pl (Y.R.S.); katarzyna.greber@gumed.edu.pl (K.E.G.); 2 Laboratory of Environmental Chemoinformatics, Faculty of Chemistry, University of Gdansk, Jana Bazynskiego 8, 80-309 Gdansk, Poland; karolina.jagiello@ug.edu.pl

**Keywords:** biomimetic chromatography, developmental neurotoxicity, New Approach Methodologies, lipophilicity, plasma protein binding, immobilized artificial membrane

## Abstract

Developmental and adult neurotoxicity are hard to assess with conventional in vitro assays, which rarely account for how a chemical actually distributes once inside the body. Biomimetic chromatography offers a practical way to fill that gap, using stationary phases that mimic phospholipid membranes and major plasma proteins to yield experimental descriptors of lipophilicity, membrane affinity, and protein binding without needing radiolabeled compounds or large sample amounts. Working within the Partnership for the Assessment of Risk from Chemicals, we profiled 67 neurotoxicology-relevant chemicals on immobilized artificial membrane, human serum albumin, and alpha-1-acid glycoprotein columns alongside lipophilicity measured at three pH values. The chromatographic lipophilicity scale matched literature log *p* values closely. Principal component and hierarchical cluster analyses of the seven descriptors pointed to one dominant hydrophobicity axis and a smaller, second axis tied to ionization and albumin binding. The panel’s overall chromatographic profile did not separate neurotoxic from non-neurotoxic reference compounds, but the most membrane- and protein-avid compounds were disproportionately positive controls and warrant closer attention. The resulting dataset gives New Approach Methodologies a ready, experimentally grounded input for in vitro-to-in vivo extrapolation and baseline toxicity assessment.

## 1. Introduction

The assessment of chemical neurotoxicity represents one of the most pressing and complex challenges in contemporary regulatory toxicology. Both adult neurotoxicity (ANT) and developmental neurotoxicity (DNT) are associated with a wide spectrum of adverse outcomes, from subtle cognitive and behavioral deficits to severe neurological disorders, and their causal links to widespread environmental chemical exposures have become increasingly difficult to dismiss. A growing body of evidence implicates numerous man-made chemicals in the disruption of complex biological processes underpinning neurological function, including endocrine signaling pathways, particularly thyroid hormone homeostasis, which is essential for normal brain maturation, as well as neuroimmune interactions such as neuroinflammation [1,2,3,4]. The growing scale of this concern reflects both the continued expansion of chemical commerce, with tens of thousands of substances in current use and limited toxicological characterization, and mounting epidemiological evidence linking early-life exposures to neurodevelopmental disorders, including autism spectrum disorders, attention-deficit or hyperactivity disorder, and learning disabilities [4,5,6,7].

Developmental neurotoxicity is particularly challenging to assess because brain development relies on a tightly coordinated sequence of processes, including neural progenitor proliferation, neuronal migration and differentiation, synaptogenesis, myelination, and programmed cell death [8,9]. Disruption of these processes during critical development windows can alter the formation and function of neural circuits, resulting in persistent effects on cognition, motor function, sensory processing, and behavior [8,9,10]. Importantly, some developmental neurotoxic effects may not be evident at the time of exposure and may emerge only later, when the affected neural systems become functionally engaged [8,10]. This delay between exposure and the emergence of measurable outcomes, together with the complexity of the developing nervous system, makes hazard identification difficult and highlights the limitations of conventional toxicity testing approaches for detecting developmental neurotoxicity [11,12].

Historically, the neurotoxic potential of chemicals has been assessed primarily using in vivo animal studies, including standardized regulatory protocols such as OECD Test Guidelines 424 for neurotoxicity studies in rodents and 426 for developmental neurotoxicity [13,14]. These studies provide information on dose–response relationships, behavioral and neuropathological effects, and susceptibility during different stages of development. However, they require substantial time, resources, and numbers of animals, particularly in the case of DNT studies [15]. Their relevance to human health may also be affected by interspecies differences in toxicokinetics, metabolism, blood–brain barrier properties, and the sensitivity of molecular targets [16,17,18].

Scientific, practical, and ethical concerns surrounding animal testing have continued to increase the support for the principles of Replacement, Reduction, and Refinement, first introduced by Russell and Burch in 1959 [19]. In the European Union, those principles are embedded in Directive 2010/63/EU, while regulatory initiatives under REACH and the Chemicals Strategy for Sustainability encourage the development and use of non-animal methods where scientifically appropriate. Similar efforts are underway internationally, including initiatives by the U.S. Environmental Protection Agency to expand the use of alternative approaches and reduce reliance on vertebrate animal testing. Together, these developments have increased the demand for methods that are both mechanistically informative and sufficiently reliable for regulatory decision-making.

In response, New Approach Methodologies (NAMs) have expanded considerably over the past decade [20,21]. Current approaches include cell-based assays using primary or induced pluripotent stem cell-derived neural models, high-content imaging, transcriptomic and metabolomic analyses, adverse outcome pathway frameworks, and microphysiological systems designed to reproduce selected features of neural tissue or the blood–brain barrier. These methods can provide valuable information on molecular and cellular responses while supporting higher-throughput chemical screening. Nevertheless, most in vitro NAMs do not inherently capture the absorption, distribution, metabolism, and excretion processes that determine internal exposure in humans.

The concentration of a chemical reaching the central nervous system depends on several factors, including bioavailability, plasma protein binding, tissue partitioning, metabolic transformation, and transport across the blood–brain barrier. Consequently, nominal concentrations applied in vitro cannot be assumed to correspond directly to concentrations in plasma, brain tissue, or other targets in vivo. This limits the direct use of in vitro effect concentrations in quantitative risk assessment unless they are integrated with dosimetry, toxicokinetic modeling, or in vitro-to-in vivo extrapolation approaches [22,23].

Biomimetic chromatography provides a practical means of generating experimentally derived descriptors relevant to chemical distribution. The approach uses stationary phases that reproduce selected interactions with biological membranes and major plasma proteins, including immobilized artificial membrane (IAM), human serum albumin (HSA), and alpha-1-acid glycoprotein (AGP) phases. Developed and systematically applied by Valkó and colleagues [24,25], these systems enable the rapid and reproducible determination of lipophilicity, expressed as the chromatographic hydrophobicity index (CHI) and CHI logD_7_._4_ at physiological pH, as well as phospholipid membrane affinity and plasma protein binding. Together, these descriptors provide information on the tendency of a compound to remain unbound in plasma or to partition into membrane-rich compartments.

An important advantage of biomimetic chromatography is that these measurements can be performed using small quantities of compound, without radiolabeling, and in a format suitable for the analysis of relatively large chemical sets. Although the resulting descriptors do not measure brain exposure, they provide distribution-related information that is often absent from conventional in vitro neurotoxicity assays. Brain availability is also influenced by blood–brain barrier permeability, active transport, metabolism, and systemic clearance; biomimetic chromatography data should therefore be regarded as complementary inputs rather than stand-alone predictors of the central nervous system. The utility of these methods has been demonstrated across diverse compound classes in pharmaceutical research [24,25,26], while more recent studies have explored their application to environmental contaminants and toxicological reference chemicals [27]. This supports their potential use alongside NAMs to improve the interpretation of in vitro bioactivity data [28,29].

The presented study was conducted within the framework of the Partnership for the Assessment of Risk from Chemicals (PARC). A curated panel of 94 chemicals of regulatory relevance, representing diverse structural classes and mechanisms associated with neurotoxicity, was selected from the PARC compound library for biomimetic chromatography characterization. Of these, 67 compounds were compatible with the UV-based analytical workflow and were successfully profiled using the selected chromatographic systems. All measurements were performed in a single laboratory under harmonized experimental conditions. This approach reduced variability associated with combining data generated across different laboratories, analytical platforms, and literature sources, thereby improving the internal consistency of the dataset.

## 2. Materials and Methods

### 2.1. Analytes

The compound panel was supplied by eMolecules (Zoetermeer, The Netherlands) as stock solutions in dimethyl sulfoxide (DMSO) at a concentration of 20 mM. Prior to analysis, the stock solutions were diluted with a 50:50 (*v*/*v*) mixture of mobile phases A and B to a working concentration of 1 mM. Narciclasine was supplied at a concentration of 1 mM and was therefore injected without further dilution.

The panel comprised 94 compounds selected within the PARC as substances of potential relevance to neurotoxicity assessment. The compound selection was based on the chemical set evaluated by Blum et al. [30]. Following the classification used in that study, the compounds included in the present panel were assigned to three categories: positive controls (*n* = 20), negative controls (*n* = 9), and compounds under investigation (*n* = 65). The complete list of compounds, their 2D structures, and their corresponding classifications is provided in Table S1.

### 2.2. Chromatographic Analysis

All biomimetic chromatographic experiments were performed using the protocols developed by Valkó and co-workers [27,28] and previously implemented in our laboratory [31,32]. The analyses included measurements of lipophilicity, phospholipid affinity, and plasma protein binding. Chromatographic Hydrophobicity Index (CHI) values were determined using fast-gradient elution and calibration against reference compounds. All analyses were carried out on a Prominence-i LC-2030C 3D HPLC system controlled by LabSolutions Lite software (version 5.136). Each analytical column was fitted with a guard column packed with the same stationary phase, and UV detection was performed over the wavelength range of 190–300 nm.

Phospholipid affinity was determined using an IAM.PC.DD2 column (100 × 4.6 mm; Regis Technologies, Morton Grove, IL, USA). Mobile phase A consisted of 50 mM ammonium acetate at pH 7.4, while mobile phase B was acetonitrile. The analyses were performed at a flow rate of 1.5 mL/min and a column temperature of 30 °C. The proportion of mobile phase B was increased linearly from 0 to 85% over 5.25 min, maintained at 85% until 5.75 min, and returned to 0% at 6.0 min. The column was then re-equilibrated for 1 min.

Plasma protein binding was determined using columns with immobilized human serum albumin and alpha-1-acid glycoprotein: CHIRALPAK HSA and CHIRALPAK AGP, respectively (both 50 × 4 mm, 5 µm; Daicel Chiral Technologies, West Chester, PA, USA). Mobile phase A consisted of 50 mM ammonium acetate at pH 7.4, and mobile phase B was 2-propanol. The flow rate was 1.2 mL/min, and the column temperature was maintained at 30 °C. The proportion of mobile phase B was increased linearly from 0 to 35% over 8.40 min, maintained at 35% for 3.20 min, and returned to 0% at 12.10 min. The total run time, including column re-equilibration, was 15 min.

Lipophilicity was determined using a Gemini NX-C18 column (50 × 3.0 mm, 5 µm; Phenomenex Ltd., Macclesfield, UK) under three pH conditions. Mobile phase A consisted of 0.01 M formic acid at pH 2.6 or 50 mM ammonium acetate adjusted to pH 7.4 or 10.5. The three pH values are those of the standard screening protocol and were selected so that both acidic and basic analytes are measured in their neutral form: at pH 2.6 carboxylic acids are largely un-ionized, at pH 10.5 amines are largely deprotonated, and pH 7.4 provides CHIlogD under physiological conditions. Acetonitrile was used as mobile phase B. The analyses were performed at a flow rate of 1.0 mL/min and a column temperature of 30 °C. The proportion of mobile phase B was increased linearly from 0 to 100% over 6.0 min, maintained at 100% until 7.0 min, and returned to 0% over 1 min. The total run time was 9.50 min.

The reference compounds used to calibrate the IAM, HSA, and AGP columns were obtained from the following suppliers: acetanilide, butyrophenone, diclofenac, warfarin, and octanophenone from Alfa Aesar (Haverhill, MA, USA); acetophenone, indometacin, paracetamol, trimethoprim, and propranolol from Sigma-Aldrich (Steinheim, Germany); nicardipine and nizatidine from Cayman Chemical (Ann Arbor, MI, USA); and carbamazepine, heptanophenone, hexanophenone, propiophenone, and valerophenone from Acros Organics (Pittsburgh, PA, USA). Lipophilicity measurements were calibrated using the Valkó calibration mixture obtained from Bio-Mimetic Chromatography Ltd. (Stevenage, UK). Retention times and control classification are presented in Table S2.

The injection volume was 5 µL. The panel was analyzed in two measurement series per column, each series comprising one injection of every compound, so that the reported duplicate values correspond to independent series rather than to repeated injections within a single sequence. The calibration mixture was injected in duplicate at the beginning of each series, in the same way as the samples, and the values obtained for each experiment are provided in Table S3. The enantioselective separation of warfarin on the HSA column was monitored throughout the campaign. The variation in retention time between replicate measurements was below 3% for all determinations, with three exceptions on the AGP column: acetamiprid (5.99%), omeprazole (4.46%), and chlorpyrifos–oxon (3.18%). In absolute terms, these differences were small, amounting to 0.104, 0.136, and 0.143 min, respectively; the elevated relative values reflect the short retention of these compounds on the AGP column rather than poor repeatability. The calibration mix values for each experiment are provided in Table S3. The coefficients of determination of the calibration lines were 0.998 for the IAM system, 0.992 for HSA, 0.987 for AGP, and 0.984, 0.988, and 0.995 for the C18 system at pH 2.6, 7.4, and 10.5, respectively.

Experimentally determined CHI values were converted to CHI_logD_ using

(1) $${CHI}_{logD}=0.0525\times CHI-1.467,$$

CHI_logP_ was taken as the highest of the three CHI_logD_ values, that is, as the retention of the neutral species. For HSA and AGP, the results obtained can be transformed into a more informative percentage of binding (%) by applying the following equations:

(2) $$\%HSA=\;\frac{101\times 10^{{logk}_{HSA}}}{1+10^{{logk}_{HSA}}}$$

(3) $$\%AGP=\frac{101\times 10^{{logk}_{AGP}}}{1+10^{{logk}_{AGP}}}$$

Four compounds were assigned a %HSA value greater than 99.8%, corresponding to the upper limit of the calibration range, but for two distinct reasons. Ibuprofen, known for its very strong plasma protein binding, and hexachlorophene, characterized by very high lipophilicity, did not elute from the HSA column under the applied gradient conditions and had to be recovered by manually flushing the column with 40% 2-propanol; the flush was monitored at the detector and stopped immediately after the compound eluted (1–2 min), followed by 10 min re-equilibration before the sequence was resumed. As no retention time could be recorded under the standard gradient, these compounds were assigned the upper-limit value. For the two brominated diphenyl ethers (BDE-47 and BDE-99), a retention time was obtained, but it exceeded that of the most strongly retained calibration standard, so the calculated binding likewise fell beyond the calibrated range and was reported as greater than 99.8%.

### 2.3. Data Analysis

All data processing, statistical analyses, and visualization were performed using the open-source statistical software R, version 4.5.1. Experimentally determined chromatographic lipophilicity (CHI_logP_) was compared with available literature octanol/water logP values. Agreement between the two measures was assessed using Pearson’s correlation coefficient (r), Spearman’s rank correlation coefficient (ρ), and ordinary least-squares regression. Differences between the two scales were further examined using Bland–Altman analysis, with the mean bias and 95% limits of agreement calculated as the mean difference ± 1.96 standard deviations.

Pairwise relationships among the chromatographic descriptors were evaluated using Pearson and Spearman correlation coefficients. Statistical significance was set at *p* < 0.05. The overall structure of the descriptor space was explored using principal component analysis (PCA) and hierarchical cluster analysis (HCA). The analyses included seven chromatographic descriptors: CHI**_logP_**, CHI_logD 2_._6_, CHI_logD 7_._4_, CHI_logD 10_._5_, CHI_IAM_, log*k***_HSA_**, and log*k***_AGP_**. Before both analyses, the descriptors were mean-centered and scaled to unit variance.

PCA results are presented as a biplot showing compound scores and descriptor loadings, with compounds colored according to their control classification. HCA was performed using Euclidean distance and Ward’s minimum variance method (Ward.D2) for both compounds and descriptors. The results are displayed as a heatmap with row and column dendrograms, each divided into two clusters. The compound dendrogram was additionally annotated according to control classification. PCA figures were prepared using the ggplot2 package (version 4.0.0), whereas the HCA heatmap was generated using the pheatmap package (version 1.0.13).

## 3. Results and Discussion

Reliable biomimetic chromatographic profiles were obtained for 67 of the 94 compounds included in the panel. Of these, 11 were positive controls, 8 were negative controls, and 48 were compounds under investigation, compared with 20, 9, and 65, respectively, in the original panel. The remaining 27 compounds could not be characterized under the applied UV-based conditions. The panel had been defined within PARC on toxicological grounds, and all its members were therefore submitted to analysis irrespective of whether their structures suggested compatibility with the chromatographic workflow. In most cases, this was due to the absence of a suitable UV chromophore or insufficient absorbance for reliable peak detection. The affected compounds included several organometallic and inorganic substances, namely triethyltin bromide, tributyltin chloride, trimethyltin chloride, manganese(II) chloride, and lead diacetate. Reliable signals were also not obtained for several small and highly polar compounds, including glycine, GABA, L-glutamic acid, glycerol, diethylene glycol, sodium valproate, hydroxyurea, and 2-amino-5-phosphonovaleric acid. Perfluorooctanoic acid and potassium perfluorohexanesulfonate likewise lacked sufficient UV absorbance under the applied conditions.

Several compounds could not be characterized for other reasons. Aspartame, despite containing an aromatic chromophore, was essentially unretained and eluted close to the column void volume, preventing the determination of a meaningful retention value. Digoxin, picrotoxin, and cyclopamine showed UV absorbance that was too weak for confident peak assignment. The complete set of chromatographic descriptors obtained for the 67 characterized compounds is presented in Table 1. Of the 27 compounds that could not be profiled, 9 were positive controls, 1 was a negative control, and 17 were compounds under investigation. Since UV detectability requires a chromophore, and chromophore-bearing compounds tend to be aromatic and hence more lipophilic, the characterized set is enriched in aromatic, mid- to high-lipophilicity compounds and depleted in ionic, organometallic, and highly hydrophilic ones. The descriptor ranges and correlation structure reported below are therefore conditional on this chemical space, and the dominance of a single hydrophobicity axis is in part a property of the compounds that could be measured. The limitation is methodological rather than intrinsic, since biomimetic descriptors have been determined for perfluorinated substances using detection independent of UV absorbance [28], and extending the workflow to mass spectrometry would recover most of the excluded compounds.

The resulting dataset covered a broad physicochemical range and included complementary measures of lipophilicity, phospholipid affinity, and plasma protein binding. Lipophilicity was examined first because it is a major factor influencing passive membrane diffusion, tissue distribution, and bioaccumulation. The measured CHI_logP_ values ranged from approximately −2.3 for the least lipophilic compounds to 6.4 for the most lipophilic ones, confirming that the study panel represented a wide range of lipophilicity.

**Figure 1:**
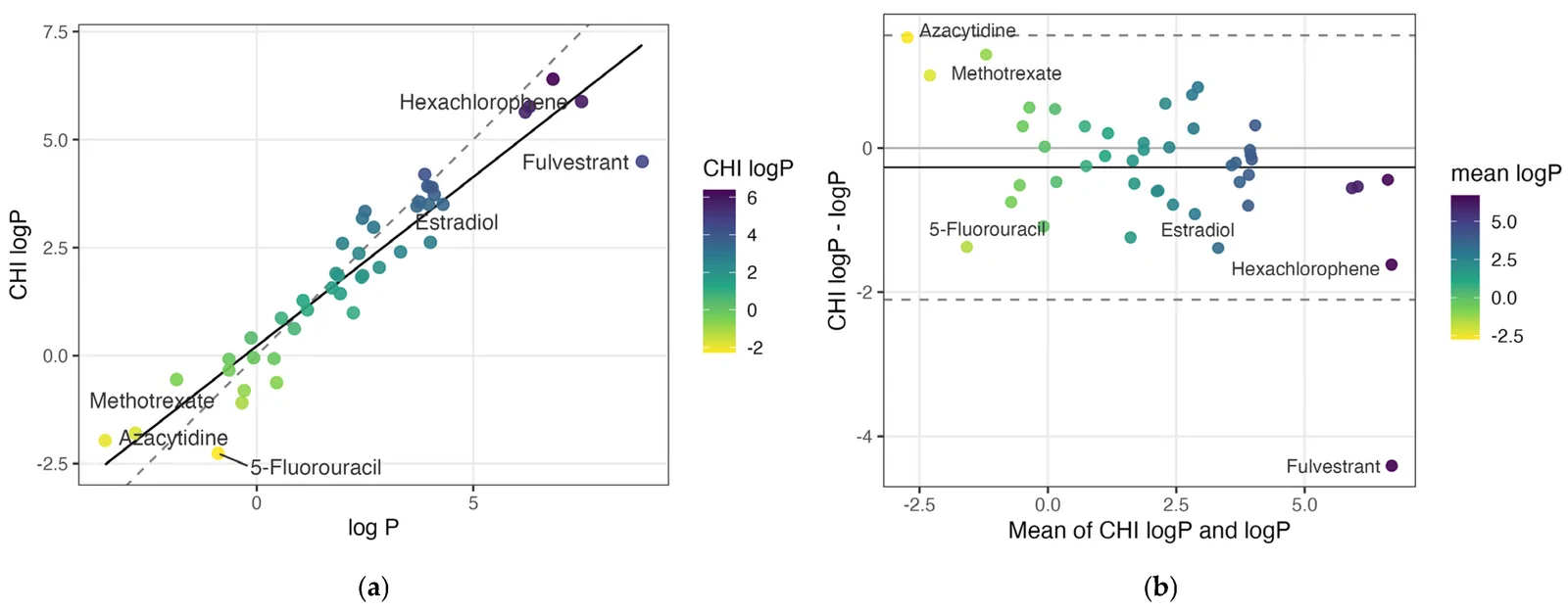
Comparison of chromatographic lipophilicity (CHI_logP_) with literature logP values for 45 compounds: (**a**) CHI_logP_ versus literature logP; dashed line, line of identity; solid line, linear regression (r = 0.94, R^2^ = 0.881). (**b**) Bland–Altman plot; solid line, mean bias (−0.27); dashed lines, 95% limits of agreement (−2.10 to 1.56). The six largest deviations are labeled.

Literature octanol/water logP values were available for 45 of the 67 characterized compounds. For this subset, CHI logP showed a strong relationship with literature logP, with a Pearson correlation coefficient of r = 0.94 (*p* = 1.6 × 10^−21^) and a Spearman rank correlation coefficient of ρ = 0.96. Because the CHI_logP_ scale is anchored to logP through its calibration, such agreement is partly built in, and the main added value is the provision of experimental data where predictions were unavailable. A closer inspection nonetheless revealed a small but systematic difference at the extremes: the chromatographic scale was slightly compressed relative to literature logP, with the most lipophilic compounds reading somewhat lower (e.g., hexachlorophene, CHI_logP_ 5.9 vs. logP 7.5) and the most hydrophilic reading somewhat higher (e.g., azacytidine, CHI_logP_ −2.0 vs. logP −3.5; methotrexate, CHI_logP_ −0.6 vs. logP −1.9), while deviations in the mid-range were negligible (mean bias −0.27; Figure 1). This mild narrowing at both ends is a recognized characteristic of gradient chromatographic lipophilicity scales and does not affect the compound ranking (ρ = 0.96) on which the subsequent analysis relies.

Having established the descriptor set, we next examined how the chromatographic parameters relate to one another. Each covered a considerable range: CHI_IAM_ extended from about −9 to 73, and plasma protein binding spanned almost the full scale, from roughly 6% to over 99.8% for HSA and from about 2% to 96% for AGP, placing the panel anywhere between compounds that are essentially free in plasma (e.g., azacytidine) and those that are almost completely bound (hexachlorophene, the brominated diphenyl ethers). Because both membrane partitioning and protein binding are driven largely by lipophilicity, a fairly tight inter-correlation among the descriptors was to be expected, and this is indeed what we found: CHI_IAM_ correlated with CHI_logP_ at r = 0.94 and with logk_AGP_ at r = 0.92, while logk_HSA_ and logk_AGP_ were related at r = 0.84. None of these correlations approach unity, however, and that shortfall is itself informative: it shows that membrane and protein retention are not simply standing in for bulk lipophilicity but capture something specific to the interaction with the phospholipid head groups and the protein binding pockets. The biomimetic phases therefore add complementary information about the compounds, information that lipophilicity alone cannot supply.

**Figure 2:**
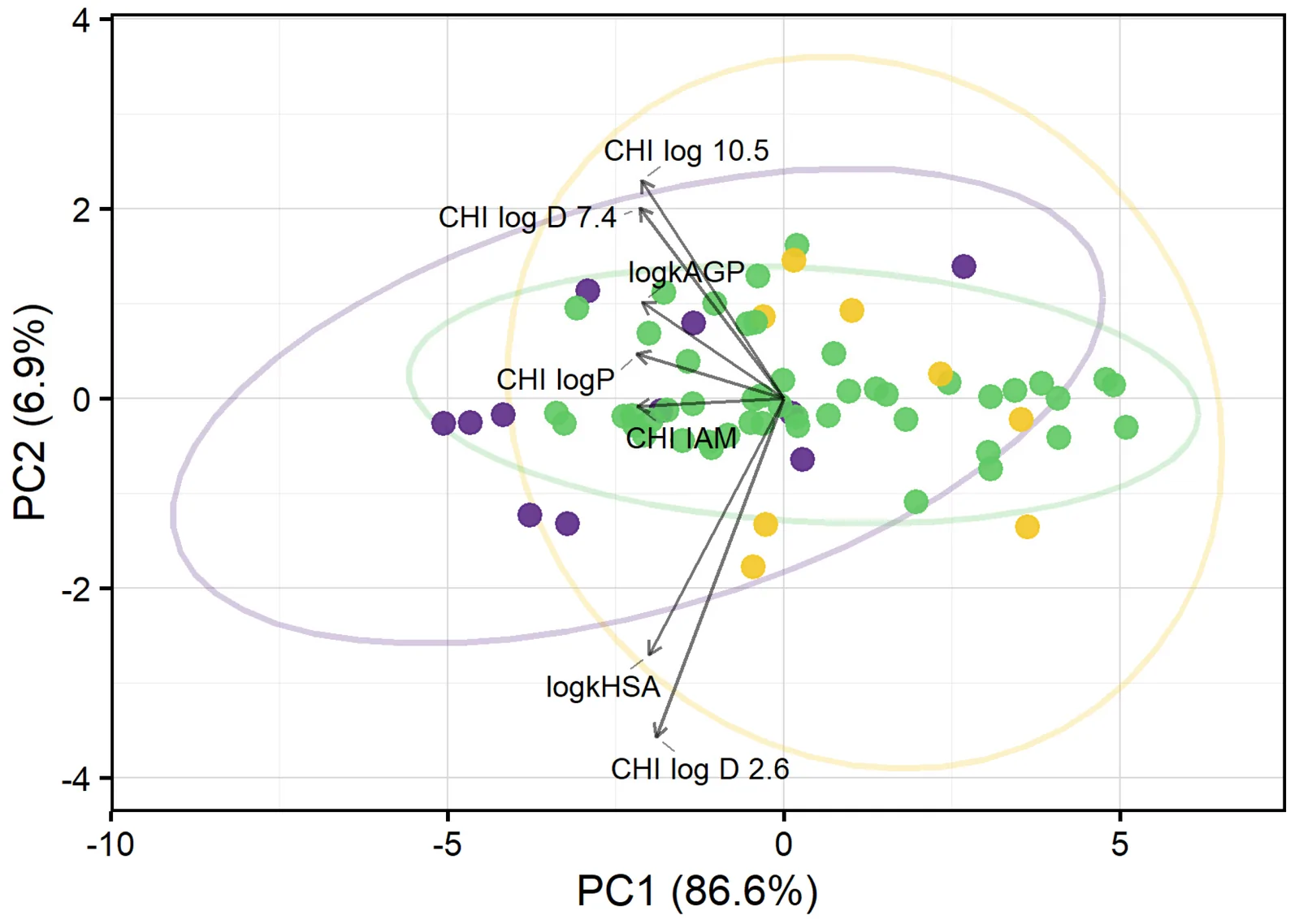
Principal component analysis of the seven chromatographic descriptors (CHI_logP_, CHI_logD 2_._6_, CHI_logD 7_._4_, CHI_logD 10_._5_, CHI_IAM_, logk_HSA_, and logk_AGP_) for the 67 characterized compounds. Points represent individual compounds and are colored by color class, with purple denoting positive controls (neurotoxic), yellow denoting negative controls (non-neurotoxic), and green denoting compounds under investigation; 95% confidence ellipses are shown for each class, and arrows denote the descriptor loadings. The three groups overlap extensively across the descriptor space, indicating that the chromatographic profile does not by itself separate neurotoxic from non-neurotoxic compounds, nor does it distinguish the compounds under investigation from either reference class.

We then turned to PCA and HCA to characterize the structure of the descriptor space as a whole. The first principal component dominated the analysis, capturing 86.6% of the total variance and drawing on all seven descriptors in roughly equal measure, consistent with the strong pairwise correlations reported above and pointing to a single dominant axis, best described as overall lipophilicity together with membrane and protein affinity, that underlies most of the variation across the panel. The second component was far smaller, accounting for 6.9% of the variance, yet far from trivial, since it set logk_HSA_ and CHI_logD 2_._6_ apart from the lipophilicity descriptors measured at neutral and high pH, showing that plasma-protein binding and pH-dependent ionization behavior together form a second, largely independent source of variation that is not reducible to lipophilicity alone (Figure 2). The confidence ellipses shown in Figure 2 should be interpreted with the size of the reference groups in mind, since 11 positive and 8 negative controls define them only loosely.

**Figure 3:**
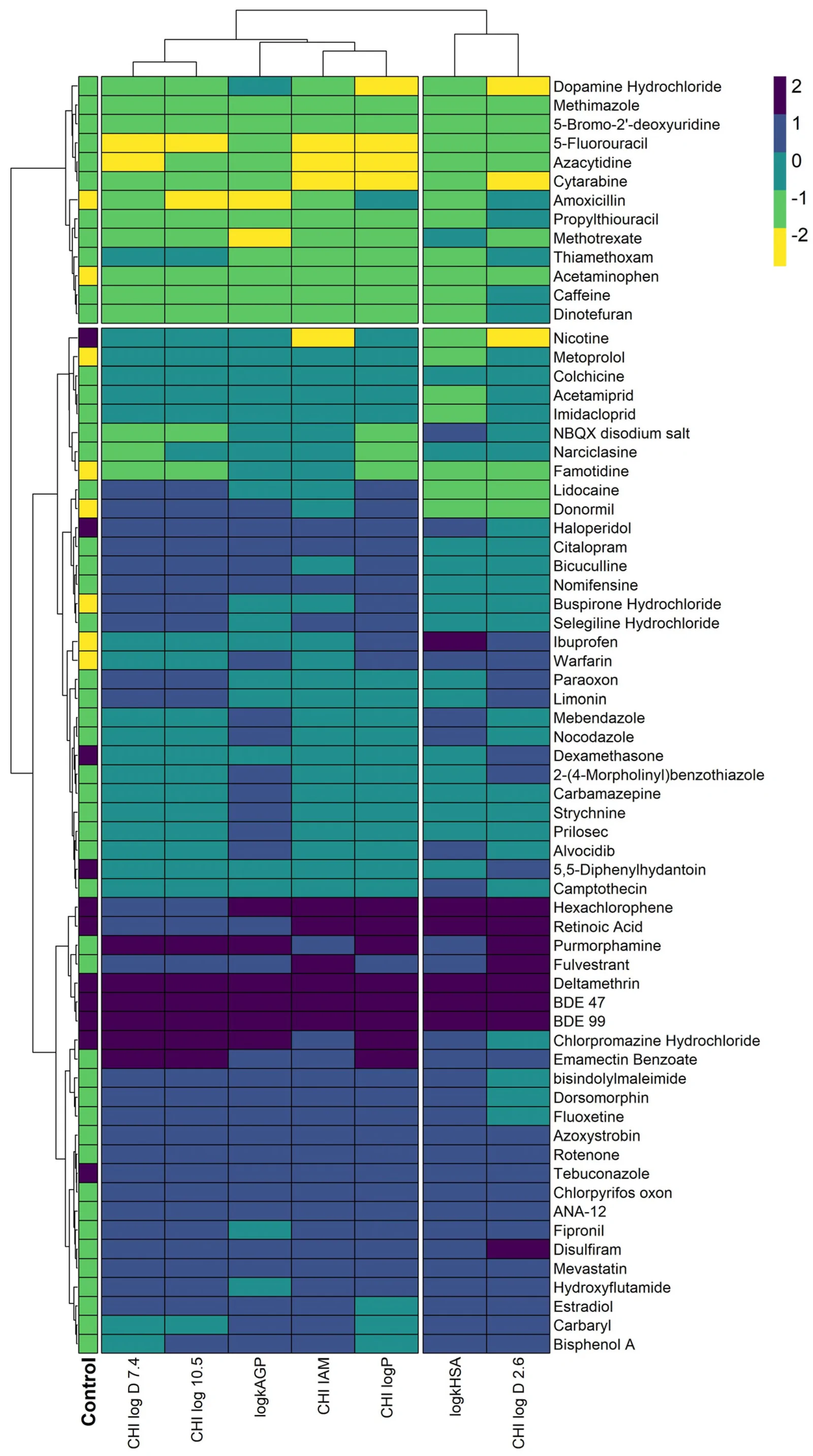
Hierarchical cluster analysis (heatmap) of the seven chromatographic descriptors across the 67 characterized compounds. Cell color encodes the standardized descriptor value, from high (dark purple) to low (yellow); rows (compounds) and columns (descriptors) are ordered by Ward’s minimum-variance (Ward.D2) clustering with Euclidean distance. The left-hand annotation bar indicates the control class, with dark purple denoting positive controls (neurotoxic), yellow denoting negative controls (non-neurotoxic), and green denoting compounds under investigation.

The hierarchical cluster analysis reproduced the same overall picture (Figure 3). Among the descriptors, logk_HSA_ and CHI_logD 2_._6_ again separated into a branch of their own, apart from the tightly grouped lipophilicity and membrane descriptors, reinforcing the earlier finding that albumin binding and acidic-pH retention are the least redundant of the seven measurements. At the compound level, the panel clustered largely by overall lipophilicity, with a hydrophilic block (dopamine, azacytidine, 5-fluorouracil, and caffeine, among others) at one end and a strongly lipophilic block at the other. Within that lipophilic extreme, positive reference substances are noticeably over-represented: the brominated diphenyl ethers, deltamethrin, hexachlorophene, retinoic acid, and chlorpromazine all group at the high-affinity end, alongside purmorphamine and fulvestrant, two compounds under investigation. We do not, however, interpret this pattern as evidence of a link between membrane and protein affinity and neurotoxic hazard, because the reference classes differ systematically in origin as well as in classification. A negative control requires demonstrated absence of neurotoxicity, and such evidence exists almost exclusively for very well-characterized substances, in practice marketed drugs for which data are available through registration and subsequent clinical use. The negative-control group is therefore composed of long-marketed medicinal substances by construction rather than by chance, and all eight characterized here are drugs. Such compounds have been shaped by the paradigms of drug discovery, being small and falling within a defined lipophilicity window, so they are displaced from the high-affinity extreme for reasons unconnected with neurotoxicity. Where the negative controls fall relative to that extreme therefore carries little information, and the apparent separation should be read as reflecting the composition of the reference set rather than a physicochemical signature of hazard. This is a structural limitation of the reference compounds available for such comparisons, not one that a larger panel would remove. Taken together, the biomimetic descriptors capture the physicochemical and distribution context of the compounds rather than neurotoxic potential as such, reinforcing their intended role as a complement to bioactivity data within an integrated, NAM-based assessment framework.

The PCA and HCA together do more than order the compounds; read side by side, they point to the physicochemical processes that each descriptor actually captures, and the way the descriptors themselves are grouped turns out to be mechanistically informative in its own right. The lipophilicity indices measured at neutral-to-basic pH, namely CHI_logP_, CHI_logD 7_._4_ and CHI_logD10_._5_, clustered together first, since for this largely neutral and basic set the ionization state changes little between pH 7.4 and 10.5 and retention stays much the same; CHI_IAM_ and log*k*_AGP_ then joined that group, both being governed predominantly by hydrophobic partitioning, with AGP in particular favoring neutral and basic lipophilic compounds. Log*k*_HSA_ and CHI_logD2_._6_, by contrast, formed a branch of their own, because both respond to ionization state and specifically to acidic character, rather than to lipophilicity alone. At acidic pH, the balance shifts, with acids moving toward their neutral, more strongly retained form while bases become protonated and less retained, so CHI_logD2_._6_ reorders the set relative to the higher-pH indices and effectively separates acids from bases; ibuprofen, warfarin, retinoic acid, and hexachlorophene, for instance, shift markedly upward, while basic compounds such as chlorpromazine and lidocaine drop. Albumin binding follows the same logic, since HSA offers dedicated high-affinity sites for anionic and acidic drugs, so those same acids bind far more strongly than their lipophilicity alone would predict. The clustering therefore reflects two largely independent contributions: a dominant hydrophobic axis shared by the lipophilicity, membrane, and AGP descriptors and a secondary, ionization-sensitive axis, weighted toward acidic compounds, carried by albumin binding and acidic-pH retention. These two descriptors report related aspects of the same property. Albumin binds through several distinct pockets with different specificities, and among these, the sites that accommodate anionic ligands mean that an acid ionized at pH 7.4 may bind more strongly than its lipophilicity alone would predict; the same acid measured at pH 2.6 is returned to its neutral form and gains retention on the reversed-phase column. The four compounds that reached the upper limit of the albumin scale illustrate this. The two brominated diphenyl ethers are not ionizable, their CHIlogD being unchanged across the pH range, and required CHIlogP values of 6.02 and 6.40 to reach that limit; ibuprofen and hexachlorophene, both acids, shift by +2.42 and +2.59 log units between pH 7.4 and pH 2.6 and reached the same limit at 3.50 and 5.88.

## 4. Conclusions

This work provides a curated, internally consistent set of biomimetic chromatographic descriptors for a neurotoxicology-relevant compound panel, comprising lipophilicity at three pH values, phospholipid membrane affinity, and human serum albumin and alpha-1-acid glycoprotein binding for 67 of the 94 selected substances, all measured in a single laboratory and a single experimental series. The measured lipophilicity scale agreed closely with literature logP where available and, importantly, supplied experimental values for the substances that lacked one, while the multivariate analysis showed that the descriptors are governed largely by a single lipophilicity axis but retain a distinct, ionization- and albumin-related component that lipophilicity alone does not capture. Two limitations are worth keeping in mind when reusing the dataset. Coverage is restricted to UV-detectable compounds, so 27 substances from the original panel could not be characterized, and the reference set is constrained by the availability of negative controls, which are necessarily well-characterized marketed drugs and therefore physicochemically restricted, so the descriptors are best read as physicochemical, distribution-relevant contexts rather than as stand-alone predictors of neurotoxic hazard. On this basis, the dataset is intended as a ready experimental input for New Approach Methodologies, with the plasma protein and membrane-affinity data feeding into in vitro-to-in vivo extrapolation and baseline toxicity assessment, and the descriptors available for integration with cell-based and in silico outputs within IATA-oriented, weight-of-evidence workflows for chemical safety assessment.

## Figures and Tables

**Table 1:** Experimentally determined lipophilicity, phospholipid affinity, and plasma protein-binding descriptors for the 67 compounds characterized in this study *.

Compound	CHI_logD 2_._6_	CHI_logD 7_._4_	CHI_logD 10_._5_	CHI_logP_	logP **	CHI_IAM_	%HSA	%AGP
5,5-Diphenylhydantoin	1.82	0.80	0.70	1.82	2.42	29.56	83.48	57.38
Tebuconazole	3.41	3.46	3.45	3.46	3.70	40.37	92.48	82.44
Hexachlorophene	5.88	3.29	3.27	5.88	7.50	72.67	>99.80	92.13
Retinoic Acid	5.76	2.87	2.84	5.76	6.30	64.43	99.47	84.76
Haloperidol	0.71	3.50	3.49	3.50	4.30	42.46	85.38	80.43
BDE-47	6.00	5.94	6.02	6.02		61.10	>99.80	95.33
BDE-99	6.37	6.32	6.40	6.40	6.84	65.28	>99.80	95.64
Deltamethrin	5.60	5.58	5.64	5.64	6.20	53.52	99.49	95.37
Omeprazole	0.34	0.99	0.90	0.99	2.23	24.49	83.86	60.12
Chlorpyrifos-oxon	3.47	3.52	3.51	3.52		36.01	92.03	79.49
Dexamethasone	1.83	1.90	1.84	1.90	1.83	29.99	70.97	40.29
Chlorpromazine Hydrochloride	1.06	5.13	5.17	5.17		51.96	96.55	91.95
2-(4-Morpholinyl)benzothiazole	1.87	2.37	2.32	2.37		28.74	82.19	67.11
Narciclasine	0.12	0.22	0.10	0.22		15.31	53.38	28.83
Acetamiprid	0.96	1.09	1.00	1.09		15.16	30.45	28.08
Paraoxon	2.51	2.59	2.56	2.59	1.98	27.03	65.11	48.02
Carbamazepine	1.76	1.86	1.79	1.86	2.45	26.99	74.43	68.27
Imidacloprid	0.76	0.87	0.77	0.87	0.57	13.89	33.16	28.39
Rotenone	3.68	3.73	3.72	3.73	4.1	37.33	95.46	77.70
Fipronil	3.90	3.89	3.89	3.90	4.00	42.26	97.01	59.26
Thiamethoxam	0.27	0.41	0.30	0.41	−0.13	7.91	11.71	9.66
Azoxystrobin	3.24	3.34	3.33	3.34	2.50	34.18	85.57	67.96
Camptothecin	1.45	1.56	1.49	1.56	1.74	25.32	84.20	55.03
Disulfiram	4.13	4.18	4.19	4.19	3.88	42.09	95.60	76.74
Mebendazole	1.67	2.04	1.98	2.04	2.83	31.85	92.42	71.91
Citalopram	0.62	3.55	3.55	3.55	3.76	38.58	68.57	70.89
Lidocaine	−0.55	3.18	3.16	3.18	2.44	25.73	17.33	47.33
Propylthiouracil	−0.07	−0.88	−1.04	−0.07	0.40	5.07	32.23	5.28
Methotrexate	−0.56	−0.64	−0.78	−0.56	−1.85	1.99	66.80	2.90
Methimazole	−1.28	−1.09	−1.25	−1.09	−0.34	−6.02	8.08	3.62
Azacytidine	−2.81	−1.97	−2.16	−1.97	−3.50	−9.43	6.36	3.53
Cytarabine	−2.83	−1.79	−1.98	−1.79	−2.80	−8.78	6.77	3.49
5-Bromo-2′-deoxyuridine	−0.81	−1.73	−1.91	−0.81	−0.29	−2.43	9.81	3.43
Colchicine	1.13	1.27	1.19	1.27	1.07	20.56	40.70	39.63
5-Fluorouracil	−2.26	−2.64	−2.85	−2.26	−0.89	−8.83	7.17	3.40
Limonin	2.56	2.62	2.59	2.62		27.40	37.44	28.80
ANA-12	3.52	3.53	3.52	3.53		46.21	97.13	87.59
Dorsomorphin	−0.50	3.88	3.89	3.89		47.31	95.02	89.28
Nocodazole	1.38	1.81	1.75	1.81		30.86	92.28	72.55
Mevastatin	3.89	3.92	3.92	3.92	3.95	43.05	95.00	81.14
Caffeine	−0.21	−0.05	−0.18	−0.05	−0.07	3.68	11.64	3.68
Bicuculline	−0.18	2.91	2.89	2.91		31.65	83.65	69.32
NBQX disodium salt	−0.12	−0.67	−0.82	−0.12		12.61	94.13	20.97
Dopamine Hydrochloride	−2.84	−1.64	−1.82	−1.64		6.11	20.00	18.13
Buspirone Hydrochloride	0.25	2.90	2.87	2.90		30.59	61.93	59.70
Carbaryl	2.25	2.37	2.32	2.37	2.36	39.48	97.12	77.41
Emamectin Benzoate	2.21	5.56	5.62	5.62		52.48	94.93	79.71
Fluoxetine	1.08	3.89	3.89	3.89	4.05	48.08	94.42	88.64
Strychnine	−0.44	1.44	1.36	1.44	1.93	21.90	63.93	62.90
Purmorphamine	4.78	4.86	4.90	4.90		50.42	98.33	91.63
Alvocidib	0.55	1.41	1.33	1.41		28.79	91.09	63.69
Bisindolylmaleimide i	0.51	2.93	2.90	2.93		44.43	96.52	90.37
Fulvestrant	4.48	4.47	4.49	4.49	8.90	56.93	98.62	91.53
Hydroxyflutamide	2.67	2.69	2.66	2.69		35.42	91.96	43.15
Estradiol	2.58	2.62	2.58	2.62	4.01	47.72	97.23	79.85
Bisphenol A	2.37	2.40	2.36	2.40	3.32	40.85	96.96	67.52
Nomifensine	−0.02	2.69	2.66	2.69		35.53	72.12	71.20
Acetaminophen	−0.63	−0.88	−1.03	−0.63	0.46	4.20	13.11	3.55
Doxylamine	−0.94	2.82	2.78	2.82		28.91	31.85	61.39
Famotidine	−1.12	−0.08	−0.21	−0.08	−0.64	13.35	29.04	32.95
Ibuprofen	3.50	1.08	0.99	3.50	3.97	24.40	>99.80	21.09
Metoprolol	−0.25	1.85	1.79	1.85	1.88	24.81	18.04	33.27
Warfarin	2.97	0.55	0.45	2.97	2.70	17.33	97.81	76.73
Dinotefuran	−0.49	−0.34	−0.48	−0.34	−0.64	0.25	7.77	3.48
Nicotine	−2.85	1.06	0.97	1.06	1.17	−9.29	13.76	22.75
Selegiline Hydrochloride	−0.43	3.34	3.33	3.34		32.77	53.85	53.72
Amoxicillin	0.62	−0.57	−4.60	0.62	0.87	6.30	23.81	1.80

* Experimental values were calculated from retention times averaged over two independent measurement series, with the replicate variation for each compound and chromatographic system reported in Table S2; ** literature values.

## Data Availability

The data presented in this study are openly available in Zenodo at https://doi.org/10.5281/zenodo.21807622.

## Supplementary Materials

The following supporting information can be downloaded at Zenodo at https://doi.org/10.5281/zenodo.21807622 (accessed on 5 August 2026).

## Abbreviations

The following abbreviations are used in this manuscript:

ANT	Adult Neurotoxicity
DNT	Developmental Neurotoxicity
NAMs	New Approach Methodologies
IAM	Immobilized Artificial Membrane
HSA	Human Serum Albumin
AGP	Alpha-1-Acid Glycoprotein
CHI	Chromatographic Hydrophobicity Index
PARC	Partnership for the Assessment of Risk from Chemicals
DMSO	Dimethyl Sulfoxide
PCA	Principal Component Analysis
HCA	Hierarchical Cluster Analysis
OECD	Organization for Economic Co-operation and Development
HPLC	High-Performance Liquid Chromatography
UV	Ultraviolet
REACH	Registration, Evaluation, Authorization, and Restriction of Chemicals
IATA	Integrated Approaches to Testing and Assessment
